# Design of image intelligent focusing system based on improved SMD function and RBF algorithm

**DOI:** 10.1371/journal.pone.0307319

**Published:** 2024-08-08

**Authors:** Qianwei Deng, Chee-Onn Wong, Roopesh Sitharan, Xiangbin Meng

**Affiliations:** 1 School of Film and Television Media, Wuchang University of Technology, Wuhan, Hubei, China; 2 Faculty of Creative Multimedia, Multimedia University, Cyberjaya, Negeri Selangor, Malaysia; 3 Cheung Kong School of Journalism and Communication, Shantou University, Shantou, Guangdong, China; Majmaah University College of Applied Medical Sciences, SAUDI ARABIA

## Abstract

The utilization of digital statistical processes in images and videos can effectively tackle numerous challenges encountered in optical sensors. This research endeavors to overcome the limitations inherent in traditional focus models, particularly their inadequate accuracy. It aims to bolster the precision of real-time perception and dynamic control by employing enhanced data fusion methodologies. The ultimate objective is to facilitate information services that enable seamless interaction and profound integration between computational and physical processes within an open environment. To achieve this, an enhanced sum-modulus difference (SMD) evaluation function has been proposed. This innovation is founded on the concept of threshold value evaluation, aimed at rectifying the accuracy shortcomings of traditional focusing models. Through the computation of each gray value after threshold segmentation, the method identifies the most suitable threshold for image segmentation. This identified threshold is then applied to the focus search strategy employing the radial basis function (RBF) algorithm. Furthermore, an intelligent focusing system has been developed on the Zynq development platform, encompassing both hardware design and software program development. The test results affirm that the focusing model based on the improved SMD evaluation function rapidly identifies the peak point of the gray variance curve, ascertains the optimal focal plane position, and notably enhances the sensitivity of the focusing model.

## 1 Introduction

The advent of imaging systems has assumed a pivotal role in the acquisition of human physical information, significantly enhancing our comprehension of the physical world. Concurrently, these systems bear immense significance in propelling the development of Cyber-Physical Systems within the optical domain. Presently, imaging systems utilizing optical devices as their primary image acquisition tools find widespread utility across agriculture, industry, military, medical, aerospace, and various other sectors. Their applications span from ubiquitous gadgets like mobile phones, tablet computers, and digital cameras to intricate Microsystems, photographic equipment, and traffic monitoring tools. They extend further to encompass sophisticated instruments such as theodolites, space telescopes, satellite imaging apparatus, and missile tracking systems [1–3].

Analogous to the human visual recognition system, the optical imaging system mirrors the function of the human eye’s retina, effectively transforming light energy to ensure visibility at varying distances. Similar to the human eye, optical lenses necessitate a zoom and focus structure. Through strategic adjustments in focal length and image distance, facilitated by zoom and focus mechanisms, these systems ensure clear imaging of targets situated at diverse distances. Consequently, zoom and focus operations stand as pivotal procedures for guaranteeing the imaging quality of cameras. Early focusing methods predominantly relied on the subjective judgment of the naked eye and manual modes, coined as manual focusing [4]. However, manual focusing, contingent upon the sensitivity characteristics of the human eye, inevitably resulted in visual errors and relatively slow focusing speeds, rendering it incapable of achieving real-time focusing. Consequently, in scenarios demanding swift scene transitions and high precision in focusing speed and accuracy, manual focusing proved inadequate. Subsequently, propelled by the rapid evolution of electronic information technology and the remarkable advancements in large-scale integrated circuit chips [5–7], imaging focusing technology has undergone a transformative shift towards intelligent and automatic methodologies. Within this trajectory, autofocus technology emerged [8,9].

Autofocus, an electronic optical system, adeptly regulates the camera lens structure throughout the entire imaging process, autonomously achieving clear and well-defined edge points without manual intervention. Early autofocus techniques predominantly encompassed focus detection and distance measurement methods, yet both methodologies suffered from drawbacks like intricate equipment and high costs [10–12]. To address the need for miniaturization and cost-effectiveness in optical imaging systems, autofocus technology pivoted towards image processing [13]. Autofocus technology leveraging digital image processing offers several advantages [14–17]. Firstly, it boasts user-friendliness and remarkable adaptability. By selecting a suitable definition evaluation algorithm to quantify image quality and formulating an appropriate search strategy to regulate the focusing motor, this technology can be flexibly applied across diverse scenes. Its minimal reliance on hardware platforms renders it highly versatile, obviating the need for hardware customization for different scenarios.

In general, large and medium-sized optical measurement equipment are susceptible to environmental factors, notably temperature, humidity, and illumination. These factors can induce alterations in certain parameters of the focusing system and even affect the imaging focal plane of optical lenses. Consequently, traditional focusing methods relying on auxiliary measurement equipment become vulnerable and necessitate extensive field adjustments to compensate and calibrate, rendering them susceptible to environmental impacts. In contrast, image-based focusing methods operate directly based on collected images, effectively circumventing the interference posed by environmental factors. This attribute allows these methods to eliminate environmental interferences, offering a robust solution for focusing unaffected by external conditions.

The presence of local extremities within the image evaluation function, influenced by external factors, poses a significant challenge. Should the search algorithm remain confined within these local extremities during the focusing process, the focusing structure might erroneously perceive the local extremum as the optimal focus. This misconception could prematurely terminate the focusing step, resulting in an inaccurate imaging system focus and, consequently, the output of a fuzzy image, undermining focusing accuracy [18,19]. Hence, studying strategies to prevent the actuator from falling into local extremities during the focusing search process holds paramount importance.

This paper addresses this challenge by leveraging neural networks to evaluate the threshold values of segmentation functions and assess each threshold value. The key innovations presented herein include:

- Designing an intelligent focusing system on the Zynq development platform, encompassing hardware design and software program development, rooted in the Cyber-Physical System paradigm.
- Proposing an intelligent focusing model integrating an RBF neural network and an enhanced SMD evaluation function. This approach enhances the SMD evaluation function by substituting the original variance sum method with the variance product method.
- Demonstrating that the intelligent focusing system developed in this study excels in achieving optimal segmentation for diverse images concerning regional consistency and contrast.

The organization of this article is as follows. Section 2 summarizes the related works; Section 3 provides a overall design of intelligent focusing system; Section 4 introduces the focus search algorithm based on RBF and improved SMD, where the realization of the method is described in detail; Section 5 describes the experiment and result analysis of the focus effect, where we discuss The effect of different methods on image segmentation. Finally, conclusion is presented in Section 6.

## 2 Related works

### 2.1 Focusing system

In the realm of automatic focusing systems for medical microscopes, Rafael et al. [20] conducted a study aimed at comparing the computational demands of diverse evaluation functions alongside their associated focusing accuracy errors. The study meticulously selected and applied 16 distinct types of focusing evaluation functions within their system. The principal objective was a rigorous scientific exploration into how noise and illumination intensity affect the performance of these evaluation functions concerning focusing accuracy. Their analysis aimed to unearth insights regarding the appropriateness and efficacy of various evaluation functions across varying conditions. Similarly, Finocchiaro [21] introduced an autofocusing system tailored explicitly for applications in Imaging and Materials Science & Engineering. Their system harnessed a focusing evaluation algorithm grounded in a contrast optimization approach. To comprehensively gauge the algorithm’s performance, they conducted both qualitative research and quantitative analysis. This thorough assessment sought to validate the algorithm’s alignment with automatic focusing systems, emphasizing its potential practical utility.

Extending the frontiers of research in automatic focusing technology, Liu et al. [22] proposed an innovative approach. Their method integrated the generalized multi-layer STOLT transfer optimization algorithm into the focusing process, aiming to heighten the precision and efficacy of automatic focusing in microscopy applications. Furthermore, J M et al. [23] introduced an autofocusing methodology for automatic optical microscopes leveraging an embedded GPU. This method amalgamated optimal focal length detection with an extended depth of field optimization algorithm. By leveraging multi-focus fusion, the method endeavored to amalgamate multiple images into a singular high-quality image. Moreover, the incorporation of an advanced NVIDIA system chip augmented the computational prowess and real-time performance of the system.

These collective studies significantly contribute to the evolution of automatic focusing systems across diverse domains, such as medical microscopy and materials science. They underscore the criticality of evaluating a spectrum of evaluation functions, introducing novel algorithms, and integrating state-of-the-art technologies to augment the accuracy, efficiency, and real-time capabilities of automatic focusing systems. Through continuous exploration in these arenas, researchers strive to bolster the quality and dependability of imaging systems used across various scientific and medical applications.

### 2.2 Image detection

Image segmentation had played a pivotal and foundational role in the realm of computer vision processing, serving as an essential prerequisite for the thorough comprehension and analysis of images. Within the context of the infrared depiction of a transformer, noticeable variations were discernible between the gray values characterizing the transformer area and the ambient background objects. The employment of the threshold method allowed for the discernment and separation of the target area from its surrounding background by precisely identifying and exploiting the discrepancies in gray values, a methodology meticulously outlined by Yan [24]. The groundbreaking work by Kapur et al. [25] heralded the introduction of the maximum entropy method, a technique that swiftly gained widespread acceptance and employment within the field of image segmentation. In this particular study, the focus was on presenting and refining a novel adaptation of the maximum entropy methodology, emphasizing the maximization of Tsallis entropy. Additionally, Pare et al. [26] presented an advancement in this area by advocating for an enhanced formulation involving a new generalized entropy, ingeniously incorporating an adjustable entropy parameter, and conclusively demonstrating its efficacy in practical application.

Within the domain of infrared image segmentation, prevalent techniques encompassed histogram-shaped threshold segmentation and clustering-based threshold segmentation, as reported by Karthick et al. [27] and Houssein et al. [28]. However, this study primarily directed its attention towards the exploration and detailed examination of the adaptive dynamic threshold selection method. Whether implementing a local threshold determined per block or tailored for individual pixels, the primary challenge observed within local dynamic threshold algorithms lay in their limitation to provide a universally applicable threshold for the comprehensive segmentation of the entire image, thus warranting further investigation and refinement in this area.

## 3 Intelligent focusing system

### 3.1 Overall design

**Fig 1:**
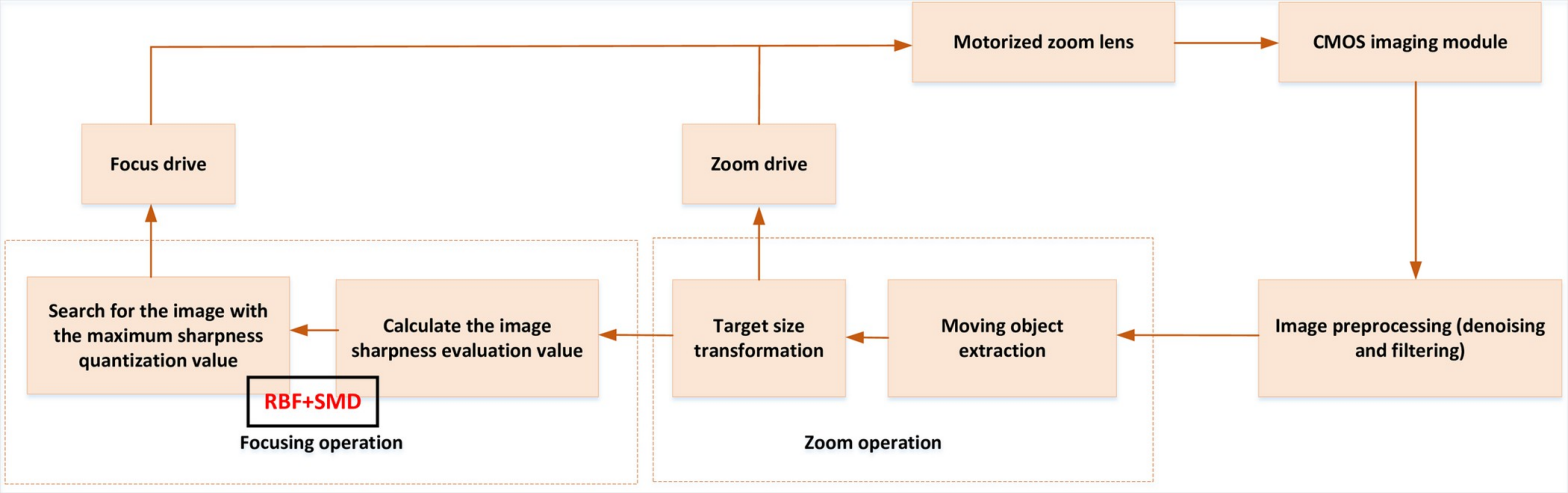
Overall system design.

The overall block diagram of the autofocus system is shown in Fig 1.

The work flow of the system is as follows:

To begin with, the CMOS imaging module plays a crucial role by capturing images and initiating the zoom stage [29]. Once the images are collected, the system analyzes them to determine whether the proportion of the target area within the image meets the predetermined value. Based on this assessment, the zoom motor is activated and mechanically adjusted to achieve the desired level of magnification. This ensures that the imaging area of the target within the field of view is appropriately adjusted for further processing.

After the zooming stage, the system proceeds to the focusing stage. Here, the quantitative value of image clarity is continuously monitored to track its change trend. The focusing motor comes into play, employing a motor search strategy to make precise adjustments. The motor is controlled in response to the observed changes in image clarity, allowing it to find the optimal focal point. This iterative process continues until the image within the field of view is deemed to be the clearest possible.

Throughout the entire process, the system enables real-time display of the collected images. These images are seamlessly transmitted to the host computer software via the network port. This feature allows for immediate visualization and analysis of the images, providing users with timely feedback on the focusing and imaging results. By facilitating real-time monitoring, the system enhances efficiency and enables users to make quick assessments and adjustments as necessary [30].

The integration of a well-defined workflow, incorporating zooming, focusing, and real-time image display, ensures that the automatic focusing system operates in a systematic and efficient manner. This enables users to capture high-quality images with optimal clarity and precision. The ability to observe and analyze images in real time further enhances the system’s usability and effectiveness, making it a valuable tool in various applications such as medical diagnostics, materials research, and scientific imaging.

### 3.2 Hardware design

**Fig 2:**
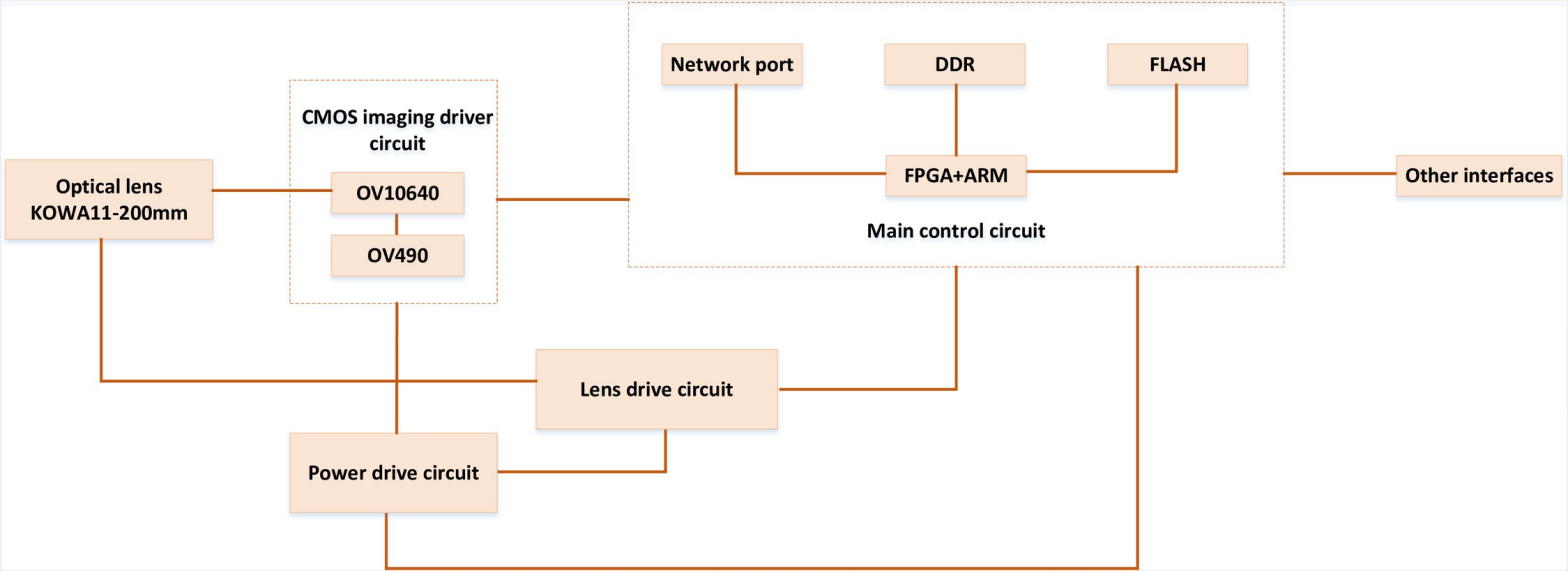
Hardware structure.

The autofocus system designed in this paper is based on FPGA + ARM platform [31]. The hardware structure diagram of the whole system is shown in Fig 2. It includes electric zoom optical lens, CMOS imaging driving circuit, main control processing circuit, power driving circuit and lens driving circuit. Output the image to FPGA+ARM processing board, according to the proportion of the target in the imaging field of view, control the movement of the zoom DC motor to complete the automatic zoom operation. Then the median filter is used to preprocess the zoomed image frame, and the evaluation value of the image frame definition is calculated by FPGA [32]. By comparing the quantization value of the adjacent frame definition, the main control circuit board sends the motor driving signal to make the DC motor drive the electric zoom lens group to move, so as to achieve the effect of autofocus.

The autofocus system designed in this paper adopts 18 times electric three variable lens, including three groups of controllable motors to connect the focal length, aperture and image distance of lens, where the optical lens is composed of a group of optical lenses, a DC motor and a feedback sensor. Moreover, two DC motors are used to control the zoom and focus functions of the lens respectively, and two groups of feedback sensors are used to feedback the focal position of the zoom ring and the focusing ring at the moment. Another motor is used to control the aperture size, which does not control the DC motor which controls the diaphragm, but directly drives the diaphragm to the maximum.

The imaging system’s image acquisition circuitry revolves primarily around the image sensor, typically categorized into CCD (charge-coupled device) and CMOS (complementary metal-oxide semiconductor) types [33,34]. The operational principle of a CMOS image sensor involves external light stimulating the pixel array, inducing the photoelectric effect, generating charge within each pixel unit, and eventually converting it into a digital image output. Noteworthy advantages of CMOS sensors include swift response, low power consumption, compact size, and cost-effectiveness. However, they are susceptible to noise, which can significantly impact image quality, resulting in noisy and uneven images.

The image processor of model 10640 comprises both an image processor module and an image sensor module. The core of the image sensor encompasses an image array, row select circuitry, column parallel analog-to-digital converter with gain control, and analog output channels. The image signal processing module manages data processing to produce the necessary output formats. Statistical data generated by the image processor contributes to automatic exposure control, automatic gain control, and automatic dynamic range control. Additionally, the processor supports functions such as lens correction, defective pixel correction, denoising, and more. Beyond these functionalities, the sensor configures internal registers through the Serial Camera Control Bus to adapt to diverse application scenarios. This flexibility allows for tailoring the sensor’s behavior and settings to suit specific needs across various imaging contexts..

### 3.3 Software design

The system uses Zynq development platform as the main control board, and the software environment is Vivado 2017.4 and XilinxSDK207.4. The PS terminal is written in C language in SDK, and PL side is modular IP in Vivado using Verilog language. The IP core is connected to Zynq kernel through block design, and the program written by PS side drives IP.

The system architecture comprises a control stream and a data stream. Data originates from the video source, entering the system via the AXI4-Stream IP core [35]. It undergoes conversion into AXI-Stream format within the video in to AXI4-Stream IP core, followed by further conversion from AXI Stream format to AXI4 Memory map format using the VDMA (video direct memory access) write channel. Ultimately, the data flows through the AXI SmartConnect IP core, connecting to the Zynq core’s AXI_HP port for reading and writing video data into DDR3 memory. The control flow encompasses clock configuration from the PS terminal to each IP core and the generation of motor driving signals. As the focusing and zooming motors operate as DC motors, only the output level of the GPIO interface necessitates configuration [36].

In order to ensure the real-time performance of image transmission, the system uses VDMA IP core provided by Xilinx for frame cache operation, and adopts three frame cache dynamic lock-in mode for caching [37]. Its workflow is that when the collected image is stored in buffer 1, the image post-processing program first processes the image frame in buffer 2; while when a new frame image is acquired, if the image of buffer 2 is being read, the new frame is alternately buffered between buffer 3 and buffer 1; After the buffer 2 reading operation is completed, the image post-processing operation is carried out for the last buffered buffer.

Therefore, the main program flow of the system software design is as follows:

1. Reset Zynq main control board and external interface equipment software, set initialization parameters, configure camera register, wait for camera to collect image;
2. Determine whether zoom processing is needed for the acquired image. If necessary, automatic zoom processing will be carried out until the target size in the collected image is appropriate;
3. Finally, the newly acquired image after zoom is automatically focused to keep the image clear.

## 4 Methodology

### 4.1 Focus search strategy based on RBF

In today’s rapid development of automation, the traditional focusing search algorithm requires the motor to move back and forth frequently to find the best focus point, and it is easy to fall into the local extreme point. Therefore, we will adopt a focusing method based on neural network algorithm to realize the auto focusing process. In RBF neural network, its hidden layer is a kind of nonlinear mapping, while the output layer is linear. The main idea of RBF is to transform data into high-dimensional space first, so as to make it linearly separable in high-dimensional space.

The structure of RBF is relatively simple. It has only three layers, i.e. input layer, hidden layer and output layer. The input layer is composed of sensing units, which can map the input vector directly to the hidden layer space without considering the weight connection. At this time, the hidden layer will carry out nonlinear transformation on the input data, The process from hidden layer to output layer will be calculated by linear transformation strategy [38].

The radial basis function is used as the activation function in the hidden layer, and the Gaussian function is usually used as the radial basis function [39]:

$$\varphi \left(∥v-u_{i}∥\right)=e^{-{\left(∥v-u_{i}∥\right)}^{2}/\sigma_{i}^{2}}i=1,2,\ldots ,n$$ (1)

In Formula (1), v is denoted as an n -dimensional input vector; u_i_ is the center of the basis function, which is a vector with the same dimension as v; σ_i_ is the normalization factor of the basis function, which determines the width of the center point of the basis function. $∥v-u_{i}∥$ indicates the distance between v and u_i_; n is the number of neuron nodes in the hidden layer.

According to the design of radial basis function, for the output layer network y(n) It can be expressed as:

$$y(n)=\sum_{i=1}^{n}w_{ik}\varphi \left(∥v-u_{i}∥\right)k=1,2,\ldots ,m$$ (2)

Where w_ik_ is the weight; k is the number of neuron nodes in the output layer. In the process of completing the autofocus algorithm, the number of nodes in the output layer will be set to 1, that is, the best focusing position point, so k can be set to 1.

According to the above formula, in the learning process, it is mainly to determine the center vector of the middle parameter u_i_, the width of σ_i_ and the weight of w_ik_. In the calculation process, the design of u_i_ is particularly important that if the center is selected too close or too far, the accuracy of the network model will be affected.

In this paper, the structure of RBF neural network is designed as follows [40]: the input layer is set as 5 nodes, the hidden layer is set as 100 neurons, and the output layer is 1 neuron, and the number of iterations is 1000. Then, according to the actual working environment of the equipment, five node parameters in the input layer of the neural network are given as a group of sample input data. The maximum value S4 of this function is calculated through the definition evaluation function, and the peak value (P4) of the focus point position at this time is recorded. P4 is the only output parameter of RBF neural network.

In order to complete the auto focusing, it is necessary to complete the establishment of the data set. Through the calculation of the image definition evaluation value at different positions of the image and the measurement of the focus position, the definition evaluation value is taken as the sample of the input layer in the RBF, and the best focal point position obtained is taken as the output. Finally, it can be used to predict the focus point. The specific steps of autofocus are as follows:

Step 1: the motor drive control platform will automatically control the chip to move vertically, so that the camera can collect four equidistant images. By calculating the images of the four equidistant points, the definition evaluation values are SS0, SS1, SS2, SS3, and the average gray values are DD0, DD1, DD2, DD3.

Step 2: Calculate the average gray value DD = DD0 + DD1 + DD2 + DD3, and then normalize the data of SS0, SS1, SS2, SS3, DD as the input of the network, and normalize it to simplify the calculation so as to improve the efficiency. After the calculation of the trained RBF model, the output layer will get the value P, and the P point coordinate is the best focus prediction position.

Step 3: The motor drive control platform will automatically control the movement until the chip moves to the P position.

Step 4: When the position of the chips have arrived to predict the best focal point p points, the electricity opportunities to control biological chip to the current position as the center and small mobile equidistant distance respectively, and calculate the evaluating value of image resolution, meet at point p focus value for maximum namely stop moving, the focus of success.

### 4.2 Improve SMD evaluation function

In the evaluation of image quality, image definition is one of the important evaluation standards of image quality, which will affect people’s ability to obtain image information. It is of great theoretical and practical value to establish an effective objective evaluation model of image clarity without reference. Image definition includes image resolution and image sharpness. The detail information of the image is represented by the image resolution, and the sharpness of the image edge change is represented by the image sharpness, where the image with higher definition generally has rich detail information, and the image edge and texture have relatively good recognition.

By dividing the gray level of the image, the image is divided into two categories according to the difference of gray value. The mathematical expression of threshold method is shown in Formular (3):

$$g(x,y)=\left\{\begin{array}{c} 255,\;f(x,y)\geq T\\ 0,\;f(x,y)<T \end{array}\right.$$ (3)

Where f(x,y) represents the input image, T represents the designed threshold, and g(x,y) represents the output image.

In this paper, the sharpness evaluation method is gray variance product evaluation function, which improves the absolute variance function of gradient evaluation function. The mathematical expression of sum of absolute values of gray difference (SMD) is shown in Formular (4)

G=|f(x,y)−f(x+1,y)|+|f(x,y)−f(x,y+1)| (4)

That is, the difference operation is performed on the gray level of the point (x,y) and its neighboring points, and the change of the gray value of the point is extracted to obtain the SMD operator.

It can be seen from Formular (4) that SMD has good computational performance, but its disadvantages are obvious, that is, the sensitivity is not high near the focus, that is, the function is too flat near the extreme point, which makes it difficult to improve the focusing accuracy. The absolute value product method of gray difference is to multiply two gray difference of each pixel field and then accumulate one by one, as shown in Formula (5)

G=|f(x,y)−f(x+1,y)|⋅|f(x,y)−f(x,y+1)| (5)

For images with separable foreground and background, there is a stable correspondence between the evaluation value of image clarity and the best segmentation threshold. to the camera autofocus principle, the focal length of the clearest image can always be found at a certain distance. In a certain range, the image to be segmented is thresholded and the image definition after thresholding is evaluated. If the segmentation threshold is near the target threshold, the definition value will be relatively stable, and the image information reflected by the definition value is relatively small. On the contrary, if it is not the target threshold, the gray variance product will fluctuate greatly.

Therefore, it is not necessary to evaluate the specific threshold value by observing the gray range of the product of gray value and line variance, and the threshold range should be evaluated first. The specific method is as follows:

1. Firstly, all gray value ranges of the image are counted, and the product of gray variance corresponding to all gray values of the interval as the threshold value is calculated, and the line graph is made;
2. By solving the curve gradient, the interval range of the stable part of the gray variance product value is found;
3. In order to determine the unique best segmentation threshold, the stable region is obtained by solving the gradient, and then the most stable part of the region is obtained by magnifying the observation of the region;
4. The gray variance product of each point in the most stationary region is calculated, and the corresponding gray value of the maximum value is selected as the segmentation threshold of the image.

## 5 Experiments and analysis

### 5.1 Dataset

We apply the Camera imaging dataset (https://zenodo.org/records/10371371) to verify our method. The dataset was detected with a camera. The height of the camera was about 4m above ground, and the camera was facing forward with an angle of 45°. This dataset consists of 4 different classes. At the end, 10,213 images have been collected.

### 5.2 Image gray level

We apply the Camera imaging dataset to verify our method. The dataset was detected with a camera. The height of the camera was about 4m above ground, and the camera was facing forward with an angle of 45°. This dataset consists of 4 different classes. At the end, 10,213 images have been collected.

**Fig 3:**
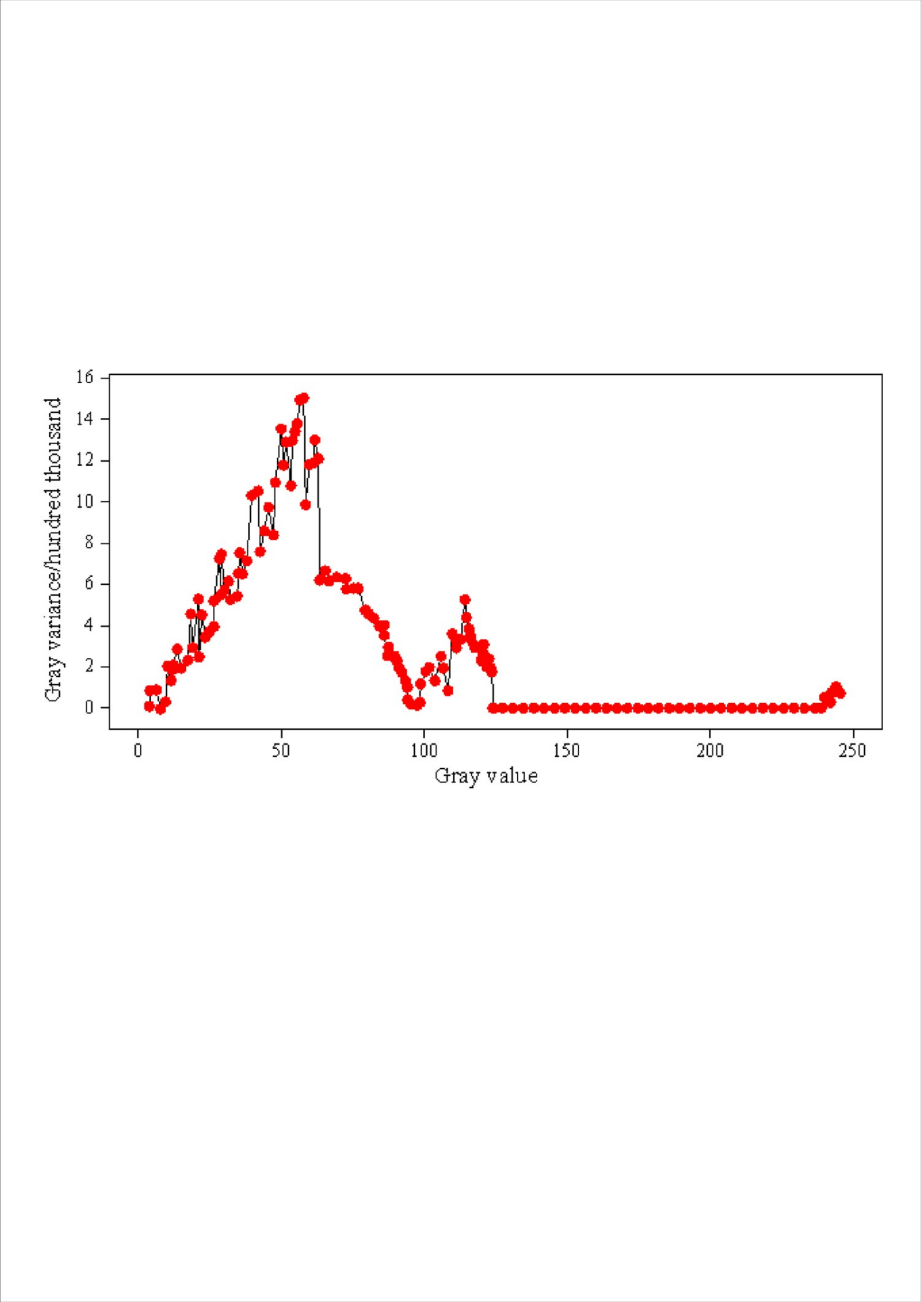
Gray variance product line chart.

After thresholding the image and calculating the product of gray variance, the discount map of the product of gray variance corresponding to each threshold is obtained, as shown in Fig 3.

Fig 3 shows a broken line graph of the product value of gray variance under all thresholds. The dot represents the product value of gray variance of the image under different thresholds. Since there is no gray value between 124 and 241 in the image, the product of gray variance in this interval is 0, which is not taken into account. In addition, it can be seen from Fig 3 that the distribution of image gray histogram is similar to that of evaluation value of product of gray variance. Both sides are approaching the gray histogram, and the product of image gray variance has a progressive process from small to large or from large to small. In addition, the line graph shows that the discount gradient in the range of image gray value increases or decreases, and there is also a small or stable gradient change, where the threshold is selected for image segmentation. After comparing with the custom threshold segmentation, it is found that the best segmentation threshold of the image is in the stable part of the sharpness evaluation broken line.

### 5.3 Comparison of focusing effect

**Fig 4:**
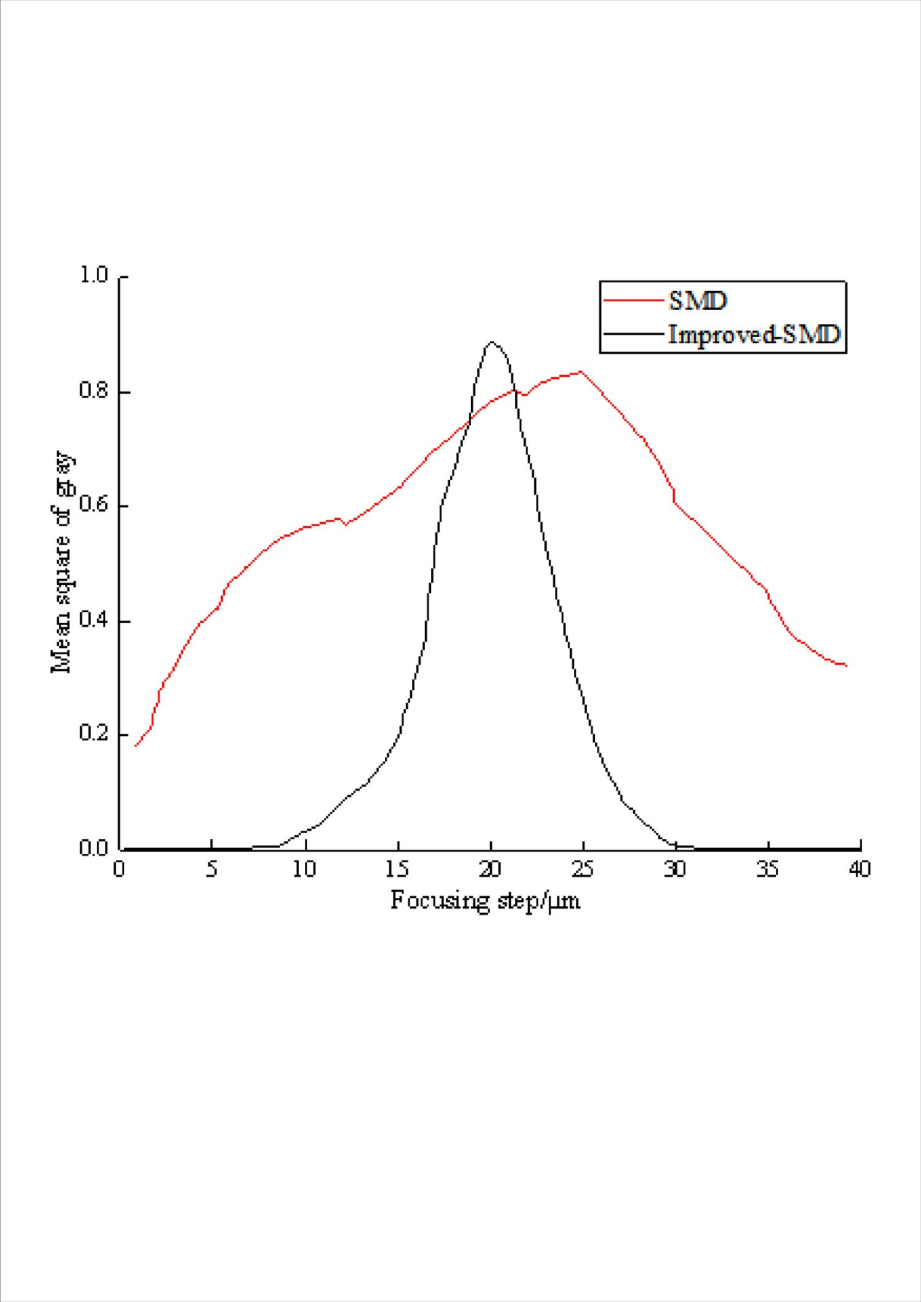
Comparison of focusing effect.

The 640×480 images captured by the camera were directly processed, and the normalized curve of gray variance function obtained from a sequence of images that changed from fuzzy to clear and then to fuzzy was shown in Fig 4.

It can be seen that there are local extremum points in the normalization curve of the original gray variance function, which has poor stability and is easy to be misjudged. Based on the improved SMD evaluation function, the model can quickly find the peak point of the gray variance curve, get the best focal plane position, and improve the sensitivity.

In this paper, region similarity index U_r_ and region contrast index C_r_ are used to objectively evaluate the experimental results. Its mathematical expression is shown in Formular (6) to Formular (8). The larger U_r_ is, the more accurate the region segmented from the image is. Where A is the sum of image gray quantities, f(x,y) is the pixel gray value of the image at (x,y), and the number of pixels in the region extracted by the corresponding segmentation algorithm of B_i_. Since this paper uses gray-processed images, the value of i is 2.


$$U_{r}=1-\left(\sigma_{1}^{2}-\sigma_{2}^{2}\right)/A$$ (6)

$$\sigma_{i}^{2}=\sum_{(x,y)\in R_{i}}{\left[f(x,y)-\mu_{i}\right]}^{2}$$ (7)

$$\mu_{i}=\sum_{(x,y)\in R_{i}}f(x,y)/B_{i}$$ (8)

When an image is segmented into non-overlapping regions, the difference degree of attributes between regions is described by the region contrast parameter shown in Formular (9). The greater the C_r_, the more accurate the segmented region will be.


$$C_{r}=\frac{\left|\mu_{1}-\mu_{2}\right|}{\mu_{1}+\mu_{2}}$$ (9)

**Fig 5:**
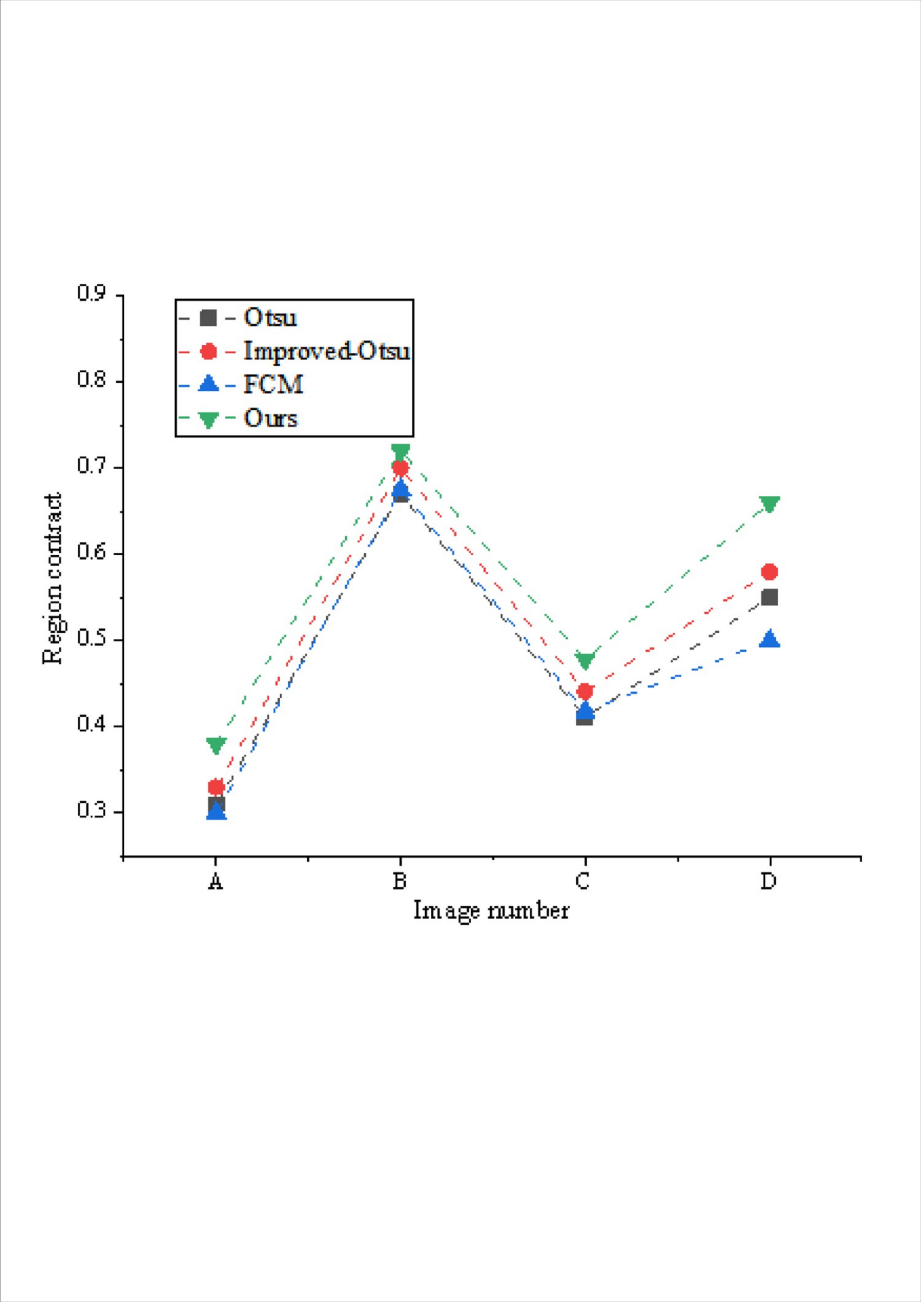
Comparison of regional contrast of different models.

**Fig 6:**
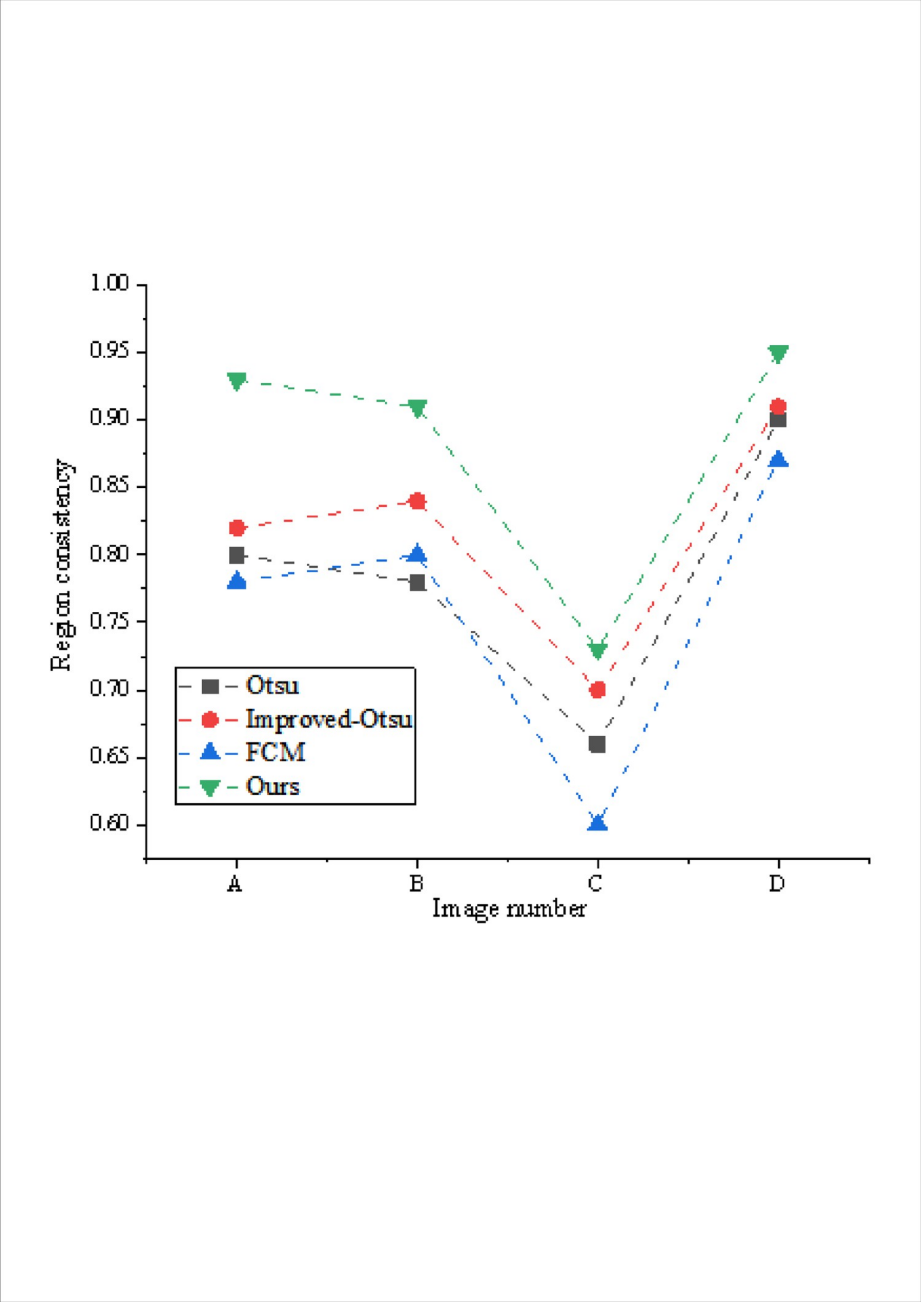
Comparison of regional consistency of different models.

The comparison of regional similarity indexes obtained by different algorithms is shown in Figs 5 and 6.

The evaluation values depicted in Figs 5 and 6 shed light on the segmentation efficacy of each algorithm across diverse images. While Otsu and its improved variant deliver substantial segmentation outcomes when the background proportion is substantial, they falter when handling scenarios with a smaller foreground, leading to significant regional contrast that fails to meet segmentation requisites. FCM showcases commendable segmentation results in certain images but struggles with ultra-low contrast scenarios. Moreover, the effectiveness of the improved method hinges on parameter selection, making it challenging to obtain suitable outcomes across different images.

A comprehensive comparison underscores that the proposed algorithm exhibits superior segmentation efficacy across varied images. Notably, the regional consistency and contrast achieved by the proposed algorithm yield optimal segmentation outcomes. This algorithm showcases a more consistent and balanced performance in handling diverse image characteristics, outperforming other methods by delivering robust segmentation results across various image compositions and contrasts.

## 6 Conclusion

The paper introduces an intelligent focusing model that synergizes RBF with an enhanced SMD evaluation function, replacing variance sum with variance product. To appraise its performance, four distinct image sets undergo simulation and analysis. The traditional Otsu algorithm, improved Otsu algorithm, FCM, and the proposed intelligent focusing model are scrutinized using three evaluation metrics: normalization curve of the gray variance function, image regional contrast, and regional consistency. These metrics offer quantitative assessments of focusing quality. The test outcomes highlight the proposed model’s exceptional performance across diverse image types. Leveraging the improved SMD evaluation function enables efficient identification of the peak point on the gray variance curve, facilitating precise determination of the optimal focal plane position. This capability enhances system sensitivity, ensuring accurate focusing. The amalgamation of RBF and the enhanced SMD evaluation function contributes to the model’s robustness and adaptability. The shift from variance sum to variance product delivers a more precise and dependable measure for assessing focusing performance. By considering multiple evaluation metrics, the model ensures comprehensive and objective assessments, delivering optimal focusing outcomes for varied images. Overall, the intelligent focusing model exhibits promising results, underscoring its potential as a potent solution for image focusing tasks. Its swift and accurate determination of the optimal focal plane position renders it invaluable across diverse domains, including microscopy, image processing, and computer vision.

## Supporting information

S1 Dataset (ZIP)
